# Immune Checkpoint Inhibitor-Induced Diabetes Mellitus: Potential Role of T Cells in the Underlying Mechanism

**DOI:** 10.3390/ijms22042093

**Published:** 2021-02-20

**Authors:** Diane Mourad, Nadim S. Azar, Assaad A. Eid, Sami T. Azar

**Affiliations:** 1 Department of Internal Medicine, Endocrinology Division, Faculty of Medicine and Medical Center, American University of Beirut, Beirut 11072020, Lebanon; diane.mourad@live.com; 2 Department of Anatomy, Cell Biology, and Physiological Sciences, Faculty of Medicine and Medical Center, American University of Beirut, Beirut 11072020, Lebanon; nadimazar1996@gmail.com (N.S.A.); ae49@aub.edu.lb (A.A.E.); 3 American University of Beirut (AUB) Diabetes, Faculty of Medicine and Medical Center, American University of Beirut, Beirut 11072020, Lebanon

**Keywords:** immune checkpoint inhibitors, immune-related adverse events, autoimmune diabetes, type I diabetes mellitus, immune checkpoint inhibitor-induced diabetes mellitus, PD-1, PD-L1, CTLA-4

## Abstract

Immunotherapy is now a recognized treatment option for several types of cancer. However, some cancer patients treated with immune checkpoint inhibitors (ICIs) are subject to immune-related adverse events, including induced diabetes mellitus. The exact role and molecular/genetic action of ICIs in diabetes are still not well understood. Elucidating the underlying mechanisms in a proper fashion would allow better refining of biomarkers that would help diagnose patients at risk of altered immune system homeostasis, but would also hold the potential of new therapeutic options for diabetes. In the present narrative review, we propose to discuss the case of autoimmune diabetes following treatment with ICIs and the role of ICIs in the pathophysiology of diabetes. We also present some scarce available data on interesting potential immune therapies for diabetes.

## 1. Introduction

Battling cancer while having to deal with an ongoing autoimmune disease is a dilemma many cancer patients treated with immune checkpoint inhibitors (ICIs) face. As immunotherapy increases the human body’s defenses, it is quite understandable to acknowledge its direct involvement in increasing the immune system’s mistakes. However, as the era of trial-and-error medicine is coming to an end and that of personalized medicine is shedding new light on how the science of the human body has always been perceived, such mishaps leading to altered immune system homeostasis are no longer admissible.

In the present narrative literature review, we will discuss the case of autoimmune diabetes following treatment with ICIs. Reviewing the available literature will allow us to gather insights into the role of immune checkpoints in the pathophysiology of type 1 diabetes mellitus (T1DM) and to propose a mechanism of pathogenesis for ICI-induced diabetes mellitus (ICIDM). Finally, we will also present some scarce available data on the involvement of immune checkpoints in the pathophysiology of other types of diabetes, such as type 2 diabetes mellitus (T2DM), and interesting potential immune therapies for diabetes.

## 2. Discussion

### 2.1. ICIs: The Rising Stars of Cancer Therapy

The past decade has witnessed the rapidly increasing notoriety of immunotherapy for the treatment of several types of cancers. Checkpoint blockade therapies affect the co-stimulatory response between tumor cells and immune cells and unleash breaks in the body’s immunity by inducing a long-lasting antitumor immune response, which would normally be suppressed. Targeting key immune checkpoints, such as programmed death-1 (PD-1) or its ligand (PD-L1) and cytotoxic T-lymphocyte antigen-4 (CTLA-4), with antibodies is now a recognized treatment option for several cancers [1].

As a matter of fact, the interaction of CTLA-4, expressed on T-cells, with its ligands CD80 and CD86 plays a vital role in the downregulation of T-cell proliferation in early responses (in lymph nodes primarily) and T regulatory (Treg) cell function, whereas the interaction of PD-1, expressed on activated T-cells (which includes Treg, B and myeloid cells), with its ligands PD-L1 and PD-L2, limits the activity of effector T cells in peripheral tissues. The body, usually kept in homeostasis, displays a balance between these complex immune regulations. However, in the case of malignancies, the balance is disrupted as some tumors expressing PD-L1 and PD-L2 interact with PD-1, promoting T-cell exhaustion associated with reduced proliferation, cytokine secretion and survival of effector T-cells, but also enhancing the proliferation of Treg with the modulation of autoimmunity on the other hand [2]. Hence, tumors can escape the human body’s immune-mediated death cells through this phenomenon [1].

ICIs are monoclonal antibodies designed to block these checkpoints, thus resulting in a de-repression of cytotoxic T cell function. In 2010, a CTLA-4-specific Immunoglobulin G1 (IgG1) monoclonal antibody, ipilimumab, was the first drug of any kind in history to provide a survival advantage in patients with advanced melanoma [3], and it was then approved by the Food and Drug Administration (FDA) in 2011 for the treatment of metastatic melanoma.

In 2014, pembrolizumab was granted accelerated approval for advanced or unresectable melanoma, rendering it the first PD-1 inhibitor cleared in the US. Moreover, the FDA granted accelerated approval for atezolizumab in 2016 to treat the most common type of bladder cancer. Atezolizumab was the first approved anti-PD-1/PD-L1 checkpoint inhibitor targeting the PD-L1 ligand as opposed to the PD-1 receptor. In 2018, two immunologists received the Nobel Prize in Medicine or Physiology in recognition of their groundbreaking contributions in the field of checkpoint immunotherapy.

Since then, new molecules and new indications for many types of cancer have been approved. To date, seven ICIs are FDA approved [4]. Table 1 summarizes these drugs.

These drugs have demonstrated an undisputable clinical benefit. However, a unique array of adverse events, called immune-related adverse events (irAEs), has been associated with ICIs and reflects their immune-based mode of action. These complications usually affect the skin, gastrointestinal tract, liver, and endocrine system [5]. A recent study showed an 11.8% incidence of endocrinopathies related to ICI use. Of those, 13.4% were due to anti-PD-1/PD-L1, 5% were due to anti-CTLA4, and 18.5% were due to sequential and/or combination treatment. The main identified endocrine complication was isolated anterior hypophysitis (6.2%) [6].

### 2.2. Autoimmune Diabetes Secondary to ICI Treatment

ICIDM has been reported with an incidence of 0.9% to 1.4% in two large case series [7,8]. This percentage increases up to 1.8% when considering patients treated with a PD-1 inhibitor (pembrolizumab and nivolumab), as no cases of ICIDM with a CTLA-4 inhibitor alone have ever been described (ipilimumab) [8]. Marchand et al. recently categorized ICIDM into four distinct entities: (a) acute diabetes with autoimmune destruction of beta cells, with most cases presenting as fulminant diabetes (characterized by an extremely rapid progression of beta-cell destruction and severe ketoacidosis, but near-normal HbA1c levels); (b) complication of autoimmune ICI-induced pancreatitis; (c) T2DM phenotype-like presentation or decompensation of previously known T2DM; and (d) diabetes following autoimmune lipoatrophy [9]. Major clinical and biological features and recommended management, as described by the authors, are summarized in Table 2.

This new classification explains some differences noted in the clinical presentation of ICIDM and allows us to better target future research towards more specific pathophysiological underlying mechanisms.

To better understand these mechanisms, we propose to review some of the major findings in the autoimmune pathway of T1DM. This will allow us to propose a hypothesis on the pathogenesis of ICIDM that remains to be validated through molecular and cellular techniques.

### 2.3. Uncovering the Autoimmune Pathway behind T1DM

T1DM is an autoimmune disease driven by CD4+ T cells that destroy insulin-secreting beta cells in the pancreas. At the early stages of insulitis, macrophages and dendritic cells infiltrate the islet and then migrate to the pancreatic lymph nodes (PLNs) in order to recruit naïve CD4+ T cells via their antigen-presenting function. In fact, these antigen presenting cells (APC) present beta cell antigens in their major histocompatibility complex (MHC) class II molecules and activate the CD4+ T cell. The differentiated T helper cells (Th1 CD4+ T) interact with APCs, and the secreted cytokines and free radicals produced induce the migration of CD8+ T cells towards the islet. The final effectors in the destruction of pancreatic beta cells are both CD4+ and CD8+ T cells [11].

The relationship between diabetes and immune checkpoint inhibitors has been widely studied during the past two decades. Although a lot has already been unveiled, the exact pathophysiological defects behind ICIDM remain largely unknown.

Preclinical studies on mice have shown that the PD-1/PD-L1 pathway is the most prominent one for the development of autoimmune diabetes. In 2003, Ansari et al. demonstrated in prediabetic non-obese diabetic (NOD) mice (used as an animal model for T1DM as they develop diabetes following insulitis) that PD-1 or PD-L1 blockade rapidly precipitated diabetes, which was not the case with PD-L2 blockade. On another hand, CTLA-4 blockade induced disease in neonates only [12]. This study was the first to highlight the important role of the PD-1/PD-L1 pathway in the regulation of T-cell activation and tolerance.

The mechanism by which this happens is still not fully understood. On the antigen-specific CD4+ T cells level, loss of PD-1 (but not PD-L1) resulted in increased cell numbers in the pancreas, spleen and pancreas-draining lymph node, increased expression of several chemokine receptors, and transition from peri-insulitis to destructive insulitis in NOD mice [13]. Consequently, PD-1 seems to regulate islet-reactive CD4+ T cells in a cell-intrinsic manner, by inhibiting their proliferation, limiting pancreas infiltration, and protecting them from diabetes [13].

However, another research study demonstrated that the total number of T cells was not significantly altered between non-diabetic wild-type mice, diabetic wild-type mice and PD-1 transgenic mice (in which expression of PD-1 was induced) [11]. In this study, it was the T cell function per se that was changed, with a noticeable reduction in FoxP3 expression in the pancreas but also the spleen of diabetic subjects in comparison to wild-type and PD-1 transgenic mice (also noting that the expression of FoxP3 in PD-1 transgenic mice was similar to that in nondiabetic wild-type controls) [11]. It is worthy of note as well that FoxP3 was found to be an important player in the suppressive function of Treg cells, and its loss or mutation leads to the development of autoimmune diseases [14]. A role for macrophages was also discussed.

Other studies have shown the protective role of PD-L1 against autoimmune diabetes. In fact, PD-L1 is expressed broadly on hematopoietic and parenchymal cells, including pancreatic islet cells, whereas PD-L2 is restricted to dendritic cells and macrophages [15]. PD-L1 expression on the parenchymal tissue (rather than on hematopoietic cells) was shown to have protective effect against autoimmune diabetes, as PD-L1 may inhibit autoreactive CD4+ T cell-mediated pathogenic tissue destruction as well as effector cytokine production [16].

PD-L1 transgenic mice were studied and a reduction in the severity of insulitis, a delay in the disease onset, and a marked reduction in the incidence of diabetes were all observed when compared to littermate controls [17]. Moreover, transgenic mice exhibited a different nature of lymphocytes, with less proliferative potential, than controls [18].

However, PD-L1 pancreatic surface expression and the frequency of PD-L1+ β cells were significantly increased as NOD mice developed diabetes with age, and this increased β cell PD-L1 expression was dependent on T- cell infiltration [19]. This could be explained by the fact that the upregulation of PD-L1 expression in insulin-producing β cells may be a protective response to limit self-reactive T cells [19]. The important role played by the pancreatic environment in islet tolerance is highlighted by another study that showed that in the absence of inflammation in the pancreas of mice, some chemotactic cytokines might be missing [20]. Insulin-specific CD4+ T cell activation status (naïve, effector or anergic) also plays a pivotal role, as the same authors had found a preferential effect of the PD-1 pathway blockade on effector but not anergic self-specific T cells. This might be supported by the finding that PD-1 is generally not expressed on naïve T cells, but rather on chronically activated T cells in peripheral tissues, particularly CD8 T cells [7].

In humans, low expression of PD-1 on activated T cells in patients with T1DM has been reported in some studies, the first of which was the study by Tsutsumi et al., in 2006 [20]. The authors noted a decrease in the PD-1 expression of CD4+ T lymphocytes in 12 T1DM patients as compared to healthy controls, highlighting that PD-1 might play a role in the development and/or maintenance of diabetes [20]. Later on, Fujisawa et al. also showed that the development of type 1 autoimmune diabetes in a Japanese population might be partly induced by a lower PD-1 expression in CD4+ T-cells through T-cell activation.

The timing of the disease evolution is crucial in understanding the underlying role of the PD-1/PD-L1 pathway in T1DM. Children with T1DM were assessed 2 weeks after diagnosis and 4–6 months post-diagnosis, while receiving insulin therapy [21]. In the former group, it was demonstrated that activated T cells fail to upregulate PD-1, as illustrated by the decreased PD-1 expression, which may be at the origin of T1DM. Nonetheless, in the latter follow-up group, the expression of PD-1 was subsequently normalized, and a significant increase in glycolysis was noted [21]. These changes might be linked to the disease evolution or even to a protective effect of exogenous insulin therapy.

The role of T cells in autoimmune diabetes and the molecular mechanisms behind it still remain unelucidated. Two studies looked into the frequency of Treg cells and their PD-1 expression in the peripheral blood of patients with long-standing diabetes under basal conditions and after T-cell CD3/CD28 stimulation.

The ratio of Treg to T effector (Teff) cells was higher in diabetic patients than that in controls, resulting from significantly higher percentages of Treg and lower percentages of Teff [22]. Treg PD-1 expression was also analyzed, and the percentages of total PD-1+, PD-1low and PD-1high expressing Treg were similar between patients and controls. After T-cell stimulation in patients, a defect in Treg cell proliferation was observed in parallel to lower percentages of total PD-1+, PD-1low and PD-1high expressing Treg [22]. The authors concluded that a novel CD8+ Treg cell population is present in T1DM patients and that it is defective due to a lower expression of PD-1 on its surface [23]. It would be interesting to further characterize this CD8+ Treg population in the pancreas and PLNs of T1DM patients.

Concerning PD-L1 expression, this ligand was found to be expressed in insulin-positive cells but absent from insulin-deficient islets in 12 T1DM patients, suggesting that beta cells upregulate PD-L1 expression in an attempt to protect themselves from the autoimmune assault. This mechanism seemed to be induced by both type I and II interferons via Interferon Regulatory Factor 1 (IRF1) [24]. This finding is supported by another study yielding similar results; a significant increase in beta cell PD-L1 expression in T1DM human samples was found in comparison to T2DM, autoantibody positive and non-diabetic samples [19]. This beta cell PD-L1 expression correlated with insulitis. On the other hand, in vitro experiments on human islets from non-diabetic individuals showed again that beta cell PD-L1 expression was promoted by Interferon-γ or IFN-γ [19].

Hence, in the context of insulitis, several pro- and anti-inflammatory cytokines, as well as other accompanying signals, are released by both immune and beta cells. Of the cytokines locally released during insulitis, two of them (namely IFNα and IFNγ) may help delay the progression of T1DM by up-regulating PD-L1 expression in human beta cells [24].

In light of all these findings, it is quite logical to propose the following mechanism for the development of T1DM: lower PD-1 expressing T cells lead to the activation of autoreactive T-cells that infiltrate pancreatic islet cells, hence contributing to the pathogenesis of T1DM [25].

### 2.4. ICIDM Proposed Pathogenesis

As for ICIDM, the inhibition of PD-1 through PD-1 or PD-L1 pharmacological blockade would theoretically lead to an increased infiltration and destruction of pancreatic beta cells by activated autoreactive T cells.

However, in this case, it is still unclear whether PD-1 inhibition leads to decreased proliferation and/or function of Treg cells with ensuing activation of autoreactive islet-specific T-cells, or if PD-1 inhibition directly removes the inhibitory pathway, thus activating these autoreactive T-cells (irrespective of Treg) [25].

**Figure 1:**
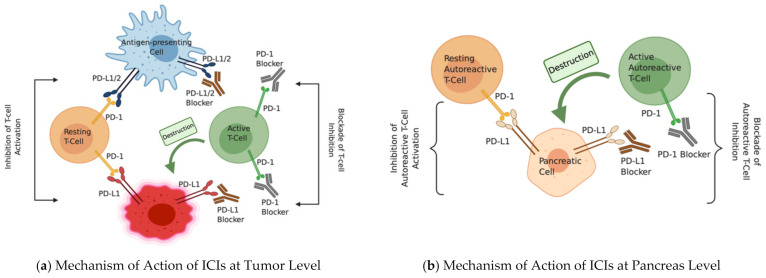
Proposed mechanism of action of ICIs at tumor and pancreas level. Proposed mechanism of action of PD-1/PD-L1 pharmacological blockade: (**a**) on the target tumor cell, and (**b**) on the pancreatic islet cell. (**a**) The antigen presenting cell (APC) presents the tumor antigen to the T cells. However, (to the left) T-cell activation in the tumor environment is inhibited by the binding of PD-1 to either PD-L1/2 on the APC or the PD-L1 on the tumor cell surface, and T cells remain in resting state. In the presence of anti-PD-1/PD-L1 antibodies (to the right), which bind and block this inhibitory pathway, T-cell activation and proliferation are both possible, and active T cells can destroy the tumor. (**b**) Since PD-L1 is also expressed by pancreatic islet cells, activation of autoreactive T-cells is inhibited via the PD-1/PD-L1 interaction (to the left). However, in the presence of anti-PD-1/PD-L1 antibodies blocking this interaction (to the right), autoreactive T-cells are activated, which leads to destruction of pancreatic islet cells and immune checkpoint inhibitor-induced diabetes. Created with BioRender.com.

Figure 1 illustrates the proposed mechanisms of action of ICIs at both the tumor and pancreas levels.

Some authors recently demonstrated that these activated autoreactive T cells respond to PD-1 blockade by producing IFN-γ, which highly activates monocyte-derived macrophages [26]. They hence gain cytocidal activity against pancreatic beta cells via nitric oxide, leading to insulinopenia and ICIDM. Nonetheless, the only report to date, to our knowledge, of the pancreatic pathology of a patient with ICIDM showed increased CD8+ T cells in and around the pancreatic islets more than CD4+ T cells, and, most importantly, an absence of macrophages [27]. While this patient presented with typical clinical and biological features of ICIDM, his T2DM history and his pancreatic metastasis render him quite unique and must lead to caution in the interpretation of the results. As such, the potential role for IFN-γ and macrophages is to be more elaborately studied, and the exact mechanism by which active autoreactive T cells destroy the pancreatic cells remains uncertain.

Future investigations should aim at elucidating the specific mechanisms of T cell activation, the function of different subtypes of PD-1-expressing T cells and the interaction between immune bodies [28]; but also, at unraveling the susceptible human leukocyte antigen (HLA) types or other genotypes, and new biomarkers representing susceptibility. Such biomarkers may help predict ICIDM, and eventually avoid it, in high risk patients. However, to this date, it still seems that HLA and genetic profiling are not always insightful. For instance, HLA DR4 and anti-glutamic acid decarboxylase (anti-GAD) antibodies were present in only 51.3% and 43.0% of cases, respectively [29]. Some authors suggest combining HLA and non-HLA-based Single Nucleotide Polymorphisms and studying these combinations in large scale studies, as the genetic landscape of ICIDM is unique [30]. For the time being, it is important to closely monitor the glucose levels and early signs and symptoms of diabetes in patients treated by ICIs, especially PD-1/PD-L1 inhibitors [29].

### 2.5. The Role of the PD-1/PD-L1 Pathway in Other Types of Diabetes

Gestational diabetes mellitus (GDM) was linked to the PD-1/PD-L1 pathway, as it appears that the downregulation of PD-1 expression on T cell subsets acts as an indicator for GDM occurrence in the third trimester of pregnancy [31]. On another level, this pathway was also associated with recovery of GDM when PD-1 expression was restored to normal [31]. Hence, PD-1 could be an interesting biomarker for GDM.

The expression of PD-1 T cells was also extensively studied in T2DM. Nonetheless, the results observed were found to be quite conflicting with the ones obtained in T1DM. One study showed increased percentages of PD-1+ CD4+ T cells and PD-1+ CD8+ T cells (suggesting increased PD-1 expression) in T2DM patients compared to healthy controls [32]. Another study confirmed this finding and also found that this proliferation response of CD4+ CD28− T-cells in T2DM was increased by advanced glycation end products [33].

Serum levels of soluble PD-L1 were also found to be much higher in T2DM than healthy individuals; in T2DM patients with atherosclerotic macrovascular diseases (ASMD), especially with acute coronary syndrome, this increase was shown to occur in an exacerbation-dependent manner [33]. The secretion of soluble PD-L1 was significantly positively correlated with the serum levels of IFN-γ and was shown to be able to enhance T-cell proliferation [33]. Hence, both the increase in soluble PD-L1 and the upregulation of PD-1 were associated with the severity of diabetic ASMD. A role for PD-L1 can be suspected in continuous T cell activation, in parallel to the IFN-γ pathway, and the development of ASMD complicating T2DM [33].

In an animal model of T2DM nephropathy, upregulation of PD-1 was also noted in peripheral blood and in kidneys [34]. This was parallel to the upregulation of systemic and local endoplasmic reticulum stress response and pro-inflammatory mechanisms, which contributes to renal injury [34]. The increased expression of PD-1 likely reflects a compensatory mechanism activated by inflammation and cell injury [34].

Finally, peripheral blood lymphocytes of T2DM patients with proliferative diabetic retinopathy (PDR) were analyzed and compared to those of age-matched patients with diabetes but no diabetic retinopathy (DM-NDR) and healthy controls [35]. The PD-1 mRNA expression was markedly decreased in the PDR group versus the other two groups. However, the frequency of PD-1+ cells was increased [35]. On another note, the expression of PD-L1 mRNA and PD-L1+ cells in the PDR group was decreased as compared to the DM-NDR and control groups [35]. This also provides insights as to the implication of PD-1 in the development of retinopathy, possibly by mediating activation-induced apoptosis.

The role of the PD-1/PD-L1 pathway in T2DM, more specifically in T2DM complications development, is still at its early beginnings and should be better characterized in respect to time and both micro- and macrovascular complications, as to really determine whether this immune pathway is linked to the pathophysiology of T2DM or only reflects a compensation mechanism in regards to T2DM inflammation and cell injury.

### 2.6. Future Perspective: The Potential for Immunotherapy in the Treatment of T1DM

As stated above, pro-inflammatory systems, induced mainly by IFNα, are put in place during insulitis, such as certain HLA class I upregulation, some specific inflammatory chemokine secretion and endoplasmic reticulum stress [36]. PD-L1 upregulation might help in counterbalancing these effects.

The blockade of IFNα appears as a good option in order to prevent the inflammation and induction of T1DM. However, it was recently shown that the blockade of type I IFNs in diabetes-prone NOD mice can prevent but also trigger the development of diabetes depending on the timing of its introduction and the stage of the autoimmune process [24].

The development of new drugs should be undertaken in such a way as to prevent IFNα-induced inflammatory chemokines, HLA class I upregulation and endoplasmic reticulum stress without inhibiting the protective PD-L1 up-regulation. The IFN-γ -JAK1/JAK2-STAT1/STAT2/STAT3-IRF1 axis, a key regulator of IFN-mediated PD-L1 expression in melanoma cells [37], seems to be a good therapeutic option.

Many challenges and barriers remain as to the clear mechanism of action by which endocrine irAEs of ICIs occur and how to counter them without compromising PD-1/PD-L1 protective actions. Nevertheless, the potential of antidiabetic therapies targeting this pathway looks very promising for T1DM, but also in regards to GDM and T2DM. Furthermore, addressing the genetics behind the interplay of immune cells may lead to the identification and early detection of patients to whom ICIs should not be indicated, as the risk of ICIDM, based on their genetic profiling, would be high.

## 3. Conclusions

The understanding of ICIDM and the mechanism of action of the PD-1/PD-L1 pathway in diabetes has dramatically increased over the last two decades. However, more research is needed to shed light on the exact role and molecular/genetic action of ICIs in diabetes, as well as the timing of PD-1 and/or PD-L1 upregulation as a protective natural reaction to inflammation and insulitis. Very few studies assessed the status of PD-1/PD-L1 expression in the pancreas and/or tumor of ICIDM patients. It would be interesting to include this analysis in further research advancements.

This will allow better refining of selected biomarkers that would help in diagnosing patients at risk of ICIDM and eventually preventing it. It also holds the potential of new therapeutic options for T1DM, with the JAK/STAT and other immune systems. Other types of diabetes are also affected by the PD-1/PD-L1 pathway and are less studied. Finding a new pharmacological target to cure, prevent or delay complications of T2DM would also be of high value.

## Figures and Tables

**Table 1:** US Food and Drug Administration (FDA) approved immune checkpoint inhibitors (ICIs) for cancer immunotherapy by chronological order.

Molecule	Brand Name	Target	Immunoglobulin (Ig) Type
Ipilimumab	Yervoy^®^	CTLA-4	IgG1
Pembrolizumab	Keytruda^®^	PD-1	IgG4
Nivolumab	Opdivo^®^	PD-1	IgG4
Atezolizumab	Tecentriq^®^	PD-L1	IgG1
Avelumab	Bavencio^®^	PD-L1	IgG1
Durvalumab	Imfinzi^®^	PD-L1	IgG1
Cemiplimab	Libtayo^®^	PD-1	IgG4

**Table 2:** Major clinical/biological features and recommended management of different categories of ICI-induced diabetes mellitus (ICIDM) [9,10].

ICIDM Category	Major Clinical/Biological Features	Recommended Management
Acute Diabetes with Autoimmune Background	Ketoacidosis (50%) or severe acute cardinal syndrome Near-normal HbA1c Undetectable C-peptide Positivity of type 1 diabetes auto-antibodies (50%) Human Leukocyte Antigen (HLA)-DR4	Multiple injections of insulin with an HbA1c target of 8.0% and lower (definitive treatment) Corticosteroid therapy is not indicated
Complication of autoimmune ICI-induced pancreatitis	Exocrine insufficiency Detectable C-peptide (early stage) Hyper-metabolism of the pancreas Possible asymptomatic pancreatitis	In early stages: basal insulin and a sulfonylurea In more advanced stages: intensive insulin therapy
T2DM phenotype-like presentation or decompensation of previously known T2DM	Increased Body Mass Index High HbA1c level Detectable C-peptide Metabolic comorbidities Medical history of type 2 diabetes	Metformin and insulin therapy (may be transitory or permanent depending on the severity of the case) Potential benefits of other antidiabetic treatments, such as Glucagon-Like Petpide-1receptor agonists or Sodium Glucose Linked Transporter 2-inhibitors, require more studies
Diabetes following autoimmune lipoatrophy	Clinical lipoatrophy Detectable C-peptide Severe Insulin resistance Non-Alcoholic Steatohepatitis Hyper-triglyceridaemia	Metformin, thiazolidinedione and multiple insulin injections

## Data Availability

No new data were created or analyzed in this study. Data sharing is not applicable to this article.
